# DNA methylation signatures can predict amyloid‐beta burden

**DOI:** 10.1002/alz.088430

**Published:** 2025-01-09

**Authors:** Michael Vacher

**Affiliations:** ^1^ Edith Cowan University, Joondalup, Australia; Australian E‐Health Research Centre, CSIRO, Perth, Western Australia Australia

## Abstract

**Background:**

A growing body of research has confirmed the presence of epigenetic alterations in Alzheimer’s disease (AD). While the causal relationship between these changes and AD remains uncertain, they offer a novel avenue to explore potential treatments. In this study, we aimed at characterising the methylation signatures of amyloid beta (Aβ) deposition, one of the main hallmarks of AD.

**Method:**

The cohort consists of 654 participants (mean age = 72.8 years (±6.83), N_(male/female)_ = 293/361) with genome‐wide DNA methylation data (Illumina Infinium EPIC 850k array), drawn from the Australian Imaging Biomarkers and Lifestyle (AIBL) study. We used a centiloid (CL) threshold of 20CL to dichotomise amyloid status of participants (Aβ^high^ N = 290, Aβ^low^ N = 364). Two methods, Partial Least Square Discriminant Analysis (PLS‐DA) and Elastic Net (EN) regression, were employed to identify methylation signatures characterising Aβ status. The overlapping set of features between the two methods were then used to build a logistic regression model to predict amyloid burden.

**Result:**

The PLS‐DA and Elastic Net models allowed the identification of 5 key methylation probes specific to low and high amyloid burden. These main features include (cg18032014 and cg17889682, DYNC1; cg06561607, SMYD3; cg03161454, NXPH4, cg06311780). Individually, all these features were significantly differentially methylated between high and low participants. Assessing the collective effect of these biomarkers in a logistic regression model resulted in 71% Area Under Curve (AUC) performance. The AUC increased to 83% when controlling for the main risk factors of AD, age and APOE ε4 genotype of participants.

**Conclusion:**

This study demonstrates the possibility to use a small set of methylation markers as a non‐invasive way to predict Aβ burden. In addition, the features identified could help shed some lights on the mechanisms underpinning Aβ accumulation. Further validation in independent cohorts is required to validate and refine the results presented in this study.

